# Supplementary material for: The Electronic Disorder Landscape of Mixed Halide Perovskites
Source: ACS Energy Lett. 2022 Nov 30;8(1):250–8. doi: 10.1021/acsenergylett.2c02352 (PMC9841609; doi:10.1021/acsenergylett.2c02352)
Supplement: Supplementary file 1 — nz2c02352_si_001.pdf [file nz2c02352_si_001.pdf]

Supplementary Information: The electronic disorder landscape of mixed halide perovskites

Yun Liu[1], Jean-Philippe Banon[2], Kyle Frohna[1], Yu-Hsien Chiang[1], Ganbaatar Tumen-Ulzii[3], Samuel D. Stranks[1,3], Marcel Filoche[2,4], Richard H. Friend[1*]

[1]Cavendish Laboratory, University of Cambridge, Cambridge, CB3 0HE, UK

[2]Laboratoire de Physique de la Matière Condensée, CNRS, École Polytechnique, Institut Polytechnique de Paris, 91120 Palaiseau, France

[3]Department of Chemical Engineering & Biotechnology, University of Cambridge, Cambridge, CB3 0AS, UK

[4]Institut Langevin, ESPCI Paris, Université PSL, CNRS, 75005 Paris, France

	$FAPbI_3$	$FAPbBr_3$
a (Å)	6.360[1]	6.013[2]
E_g (eV)	1.51[3]	2.25[3]
m_e (m_0)	0.086[4]	0.134[4]
m_h (m_0)	0.095[4]	0.128[4]
E_p (eV)	41.6[4]	39.9[4]
n	2.5[5]	2.0[5]

Table S1. Structural and electronic parameters of perovskites. The lattice parameter (*a)* and bandgaps (E_g) used in this study are based on experimental measured values of pure FA perovskites. The effective masses (m_e, m_h) and Kane's energies (E_p) are based on the computational results of the Cs counterparts, as A site cation does not participle in the band edge electronic structures and have small impact on the curvature of the bands. Average values for the real part of the refractive index n are used as their variations within the energy range investigated are small.

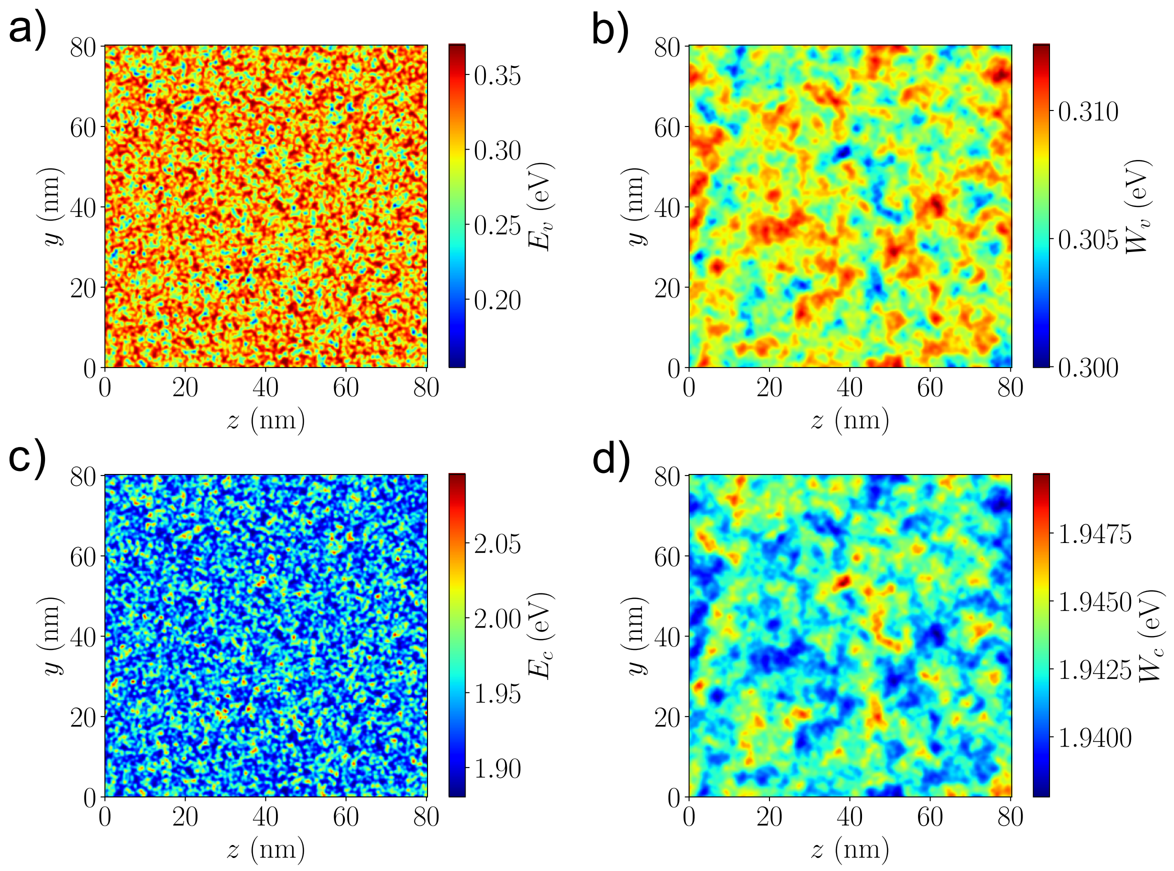

Figure S1. Local properties of $(Cs_{0.05}FA_{0.78}MA_{0.17})Pb(I_{0.83}Br_{0.17})_3$. 2D cut of the local (a) E_v and (c) E_c in the *yz* plane for whereby the I and Br atoms are randomly distributed in a simulation cube of side length of 80 nm. The energies are referenced to the VBM of the pure Br system with the band alignment scheme A (γ = 0.5). 2D cut of the respective effective confining potential (b) W_v and (d) W_c computed from the landscape equation in the same *yz* plane.

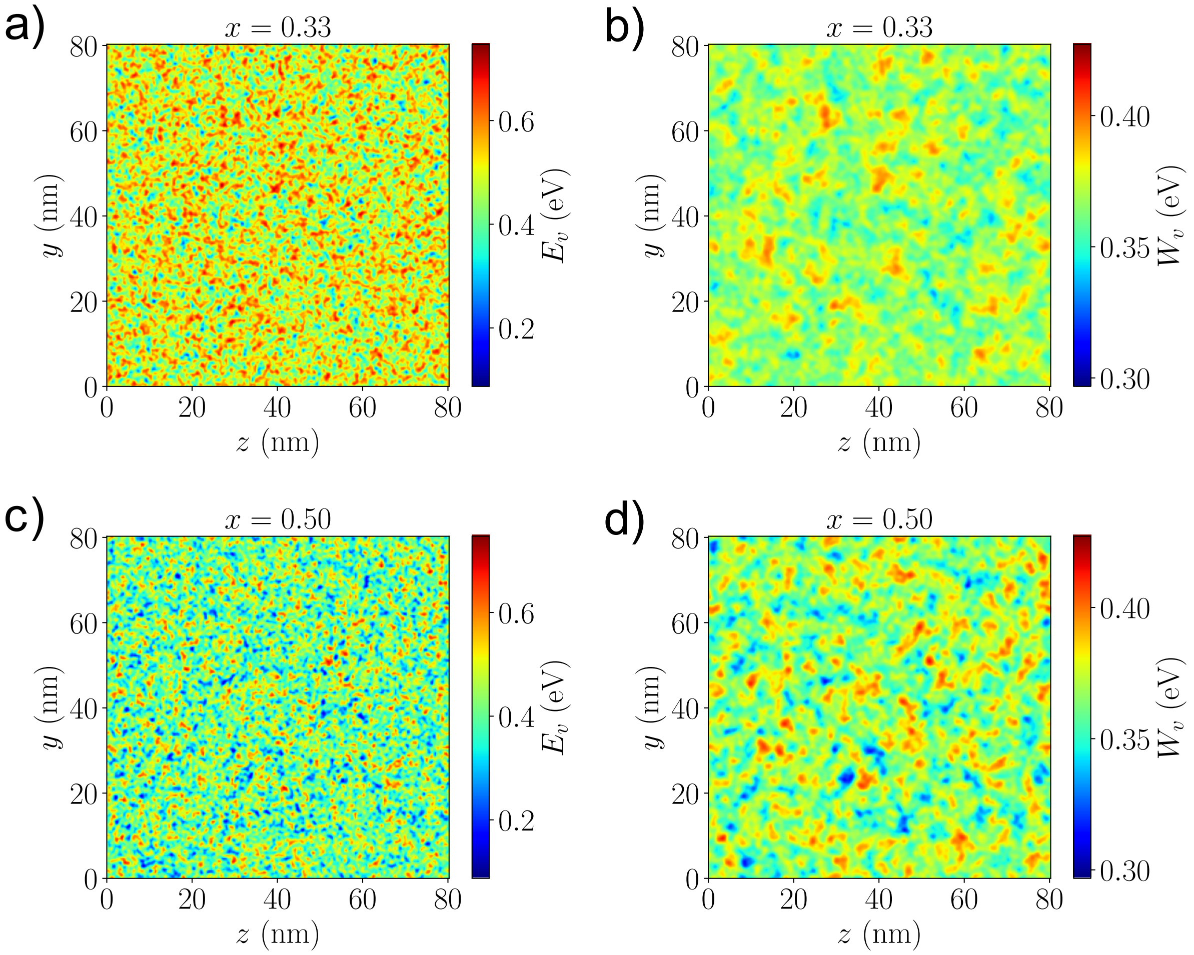

Figure S2. Local electronic structure properties of FAPb($I_{1\text{-}x}Br_x$)$_3$. 2D cut of the local (a) E_v and (c) E_c in the *yz* plane of the simulation box for $x = 0.33$ and 0.50. The energies are referenced to the VBM of the pure Br system, and band offset is entirely attributed to the valence band. 2D cut of the computed effective confining potential (b) W_v and (d) W_c from the landscape equation in the same *yz* plane.

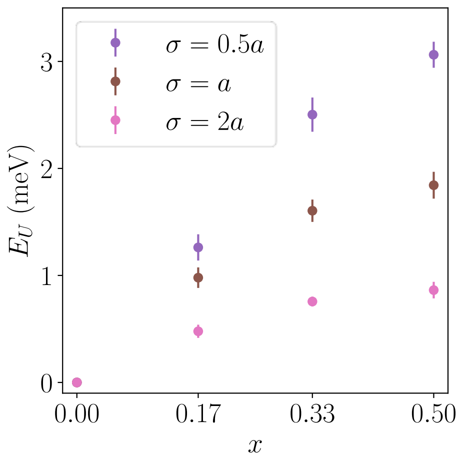

Figure S3. Urbach energy for different Gaussian smearing parameter. The extracted E_U as a function of Br concentrations with different smearing parameters σ, using band alignment scheme B (γ=0).

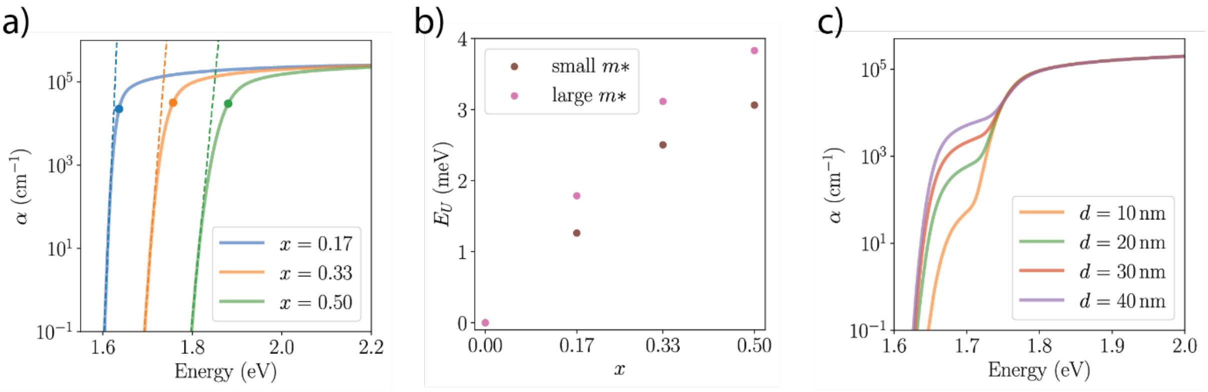

Figure S4. Absorption spectra and Urbach energy for large electron and hole effective masses. (a) The computed absorption coefficient (α) of $FAPb(Br_xI_{1-x})_3$ in logarithmic scale as a function of the photon energy, averaged over 50 independent realizations. Solid circles indicate the average E_g for each composition, and the dashed lines show the fitted exponentials below the absorption edges. Band alignment scheme B ($\gamma = 0$) and smearing parameter $\sigma = 0.5a$ are used, with effective masses twice as large as those stated in Table S1. (b) The extracted Urbach energy as a function of the Br concentrations for both effective masses used, with small values representing those as listed in Table S1. (c) The absorption spectra of the halide segregated perovskites shown in logarithmic scale, with I-rich regions of different sizes, averaged over 50 realizations. The overall composition of the simulation box is x=0.33 with I-rich regions having x=0.2.

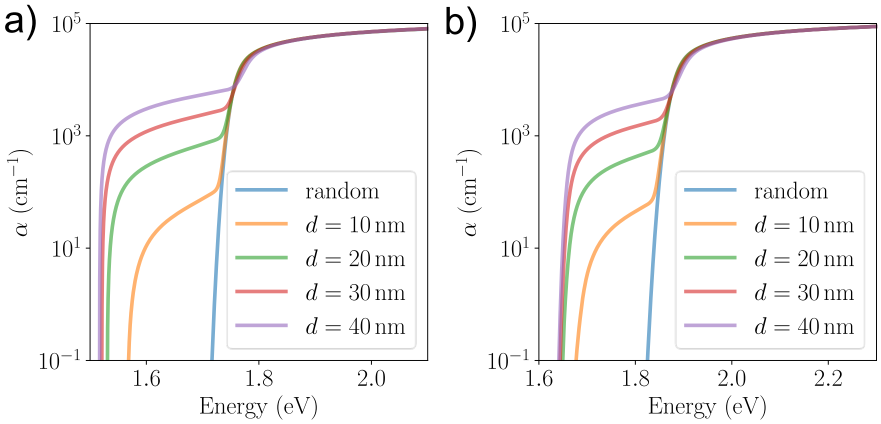

Figure S5. Absorption spectra of segregated perovskites. (a) Absorption spectra in logarithmic scale of segregated perovskite, with the I-rich region having a pure I composition (x = 0), and the overall composition of the simulation box at x = 0.33. (b) Absorption spectra in logarithmic scale of segregated perovskite, with the I-rich region with x = 0.2, and the overall composition of the simulation box at x = 0.50.

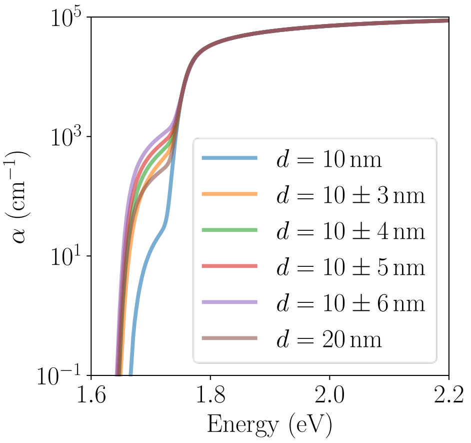

Figure S6. Absorption spectra of segregated perovskites with multiple I-rich regions.

Absorption spectra in logarithmic scale of segregated perovskite, 10 non-overlapping I-rich regions having a composition at $x = 0.2$, and the overall composition of the simulation box at $x = 0.33$. The mean diameter of the I-rich region is 10 nm with a standard deviation of 3, 4, 5 and 6 nm. These absorption spectra are compared with a single I-rich region of $d = 10$nm and $d = 20$nm. All spectra are averaged over 50 random realizations.

Reference

(1) Weller, M. T.; Weber, O. J.; Frost, J. M.; Walsh, A. Cubic Perovskite Structure of Black Formamidinium Lead Iodide, α-[HC$(NH_2)_2$]PbI_3, at 298 K. *J. Phys. Chem. Lett.* **2015**, *6* (16), 3209–3212. https://doi.org/10.1021/acs.jpclett.5b01432.

(2) Govinda, S.; Kore, B. P.; Swain, D.; Hossain, A.; De, C.; Guru Row, T. N.; Sarma, D. D. Critical Comparison of $FAPbX_3$ and $MAPbX_3$ (X = Br and Cl): How Do They Differ? *J. Phys. Chem. C* **2018**, *122* (25), 13758–13766. https://doi.org/10.1021/acs.jpcc.8b00602.

(3) Tao, S.; Schmidt, I.; Brocks, G.; Jiang, J.; Tranca, I.; Meerholz, K.; Olthof, S. Absolute Energy Level Positions in Tin- and Lead-Based Halide Perovskites. *Nat. Commun.* **2019**, *10*, 2560. https://doi.org/10.1038/s41467-019-10468-7.

(4) Becker, M. A.; Vaxenburg, R.; Nedelcu, G.; Sercel, P. C.; Shabaev, A.; Mehl, M. J.; Michopoulos, J. G.; Lambrakos, S. G.; Bernstein, N.; Lyons, J. L.; Stöferle, T.; Mahrt, R. F.; Kovalenko, M. V.; Norris, D. J.; Rainò, G.; Efros, A. L. Bright Triplet Excitons in Caesium Lead Halide Perovskites. *Nature* **2018**, *553* (7687), 189–193. https://doi.org/10.1038/nature25147.

(5) Ndione, P. F.; Li, Z.; Zhu, K. Effects of Alloying on the Optical Properties of Organic–Inorganic Lead Halide Perovskite Thin Films. *J. Mater. Chem. C* **2016**, *4* (33), 7775–7782. https://doi.org/10.1039/C6TC02135B.
